# Enhancing Tone and Strength in a Patient With Autoimmune Encephalitis and Guillain-Barré Syndrome Using Rood’s Facilitatory Techniques and Neuromuscular Electrical Stimulation: A Case Report

**DOI:** 10.7759/cureus.56054

**Published:** 2024-03-12

**Authors:** Reva Rajurkar, Nitika Chavan, Nishigandha Deodhe, Nandini C Baheti

**Affiliations:** 1 Neurophysiotherapy, Ravi Nair Physiotherapy College, Datta Meghe Institute of Higher Education and Research, Wardha, IND

**Keywords:** neuromuscular electrical stimulation, rood’s faciliatory approach, rehabilitation, guillain–barré syndrome, autoimmune encephalitis

## Abstract

This case report documents the comprehensive management of a 21-year-old female resident of Gadchiroli presenting with a 10-day history of fever, altered consciousness, and neurological sequelae following a traumatic incident. The patient exhibited a Glasgow Coma Scale score of 6/15, hypotonia in both upper and lower limbs, diminished deep tendon reflexes, and respiratory complications. This case study describes a thorough physiotherapeutic strategy that focuses on tone facilitation and muscle weakness improvement. The intervention used Rood's facilitative approaches as well as neuromuscular electrical stimulation (NMES). Rood's treatments, which emphasized mobilizing touch and tactile stimulation, brushing, quick icing, quick stretching, tapping, massaging the skin, heavy joint compression, and rolling, were used deliberately to move the patient from flaccidity to better muscle tone. These techniques' repetitive and task-specific nature coincided with motor learning principles, enabling adaptive modifications in brain networks. Concurrently, NMES was used to improve muscle activation, create a controlled environment for neurorehabilitation, and promote strength increases. The successful integration of various modalities highlights the possibility of favorable neuronal adaptations and functional improvements in individuals suffering from complicated neuromuscular disorders. This case demonstrates the need for individualized and diversified physiotherapeutic techniques in improving rehabilitation outcomes.

## Introduction

The term "autoimmune encephalitis" (AE) refers to a collection of immune-mediated, non-infectious, inflammatory illnesses of the brain parenchyma that can affect the meninges, spinal cord, white matter, or cortical or deep grey matter [1-4]. In AE, self-antigens expressed in the central nervous system (CNS) are targeted by the host immune system [5]. The most frequent known causes of encephalitis were infections before the discovery of neuroglial surface autoantibodies. This balance has been upset, nonetheless, during the past 20 years by the discovery of several autoantibodies that target the extracellular domains of neuroglial proteins in encephalitis patients [6]. In high-income nations, the annual incidence of encephalitis is estimated to be between five and 10 per 100,000 people; encephalitis affects patients of all ages and is very costly for healthcare providers, families, and society as a whole [7,8]. AE manifests as neurological and psychiatric symptoms without fever or cerebrospinal fluid (CSF) pleocytosis, as well as core symptoms similar to infectious encephalitis [9]. The majority of AE types involve mood, behavior, and memory changes, reduced consciousness, and seizures; however, the degree and predominance of certain symptoms over others, as well as the existence of additional features (such as dyskinesias, severe psychiatric manifestations, faciobrachial dystonic seizures, hyponatremia, and diarrhea), can vary [10].

Guillain-Barré syndrome (GBS) is an acute polyneuropathy mostly characterized by acute flaccid paralysis with or without sensory/autonomous nerve dysfunction [11]. It is characterized by symmetrical limb weakness and hyporeflexia or areflexia, which peaks in severity in four weeks [12]. It has an annual incidence of one to two per 100,000 people, with a higher male predominance [13]. The mortality and case fatality rates are both around 3%. During the acute period, around 25% of patients require ventilatory assistance. Although the majority of individuals recover within six months of the onset of symptoms, others experience chronic symptoms [14]. Although the clinical manifestation of the disease is varied and various discrete clinical forms occur, patients with GBS often present with weakness and sensory symptoms in the legs that proceed to the arms and cranial muscles. GBS is diagnosed based on a patient's history as well as neurological, electrophysiological, and CSF testing [15,16]. In the treatment of GBS, intravenous immunoglobulin and plasma exchange are equally beneficial; no other treatments have been proven to be effective [17].

AE with GBS refers to a rare and complex condition where the immune system mistakenly attacks the nervous system, leading to neurological impairments. GBS is characterized by the immune system attacking the peripheral nervous system, while AE involves inflammation of the brain. In some cases, there can be an overlap between these two conditions, as seen in Bickerstaff brainstem encephalitis (BBE) with GBS overlap, where patients may exhibit symptoms such as ataxia, ophthalmoplegia, altered consciousness, muscle weakness, and other neurological deficits [18,19]. The exact pathophysiology of this overlap is not entirely clear, but it is believed to involve autoimmune mechanisms and molecular mimicry triggered by infections like *Chlamydia pneumoniae* or other pathogens. Prompt recognition and appropriate management are crucial for favorable outcomes. Treatment typically involves immunotherapy with intravenous immunoglobulin and corticosteroids to modulate the immune response and reduce inflammation. While there is no known cure for GBS or AE, early intervention and supportive care can help manage symptoms and improve long-term outcomes [20].

Rood's technique is a neurophysiologic and developmental therapy approach that develops motor patterns from primitive reflexes to appropriate sensory receptors in normal sequential developmental patterns to improve motor performance. The core elements of Rood's technique are tone normalization or improvement, developmental sequence improvement, purposeful movement, and repetition or practice. Other neurophysiology-based neuro-facilitation procedures utilized by rehabilitation therapists include the Brunnstrom technique, proprioceptive neuromuscular facilitation, and neurodevelopmental therapy (also known as neurodevelopmental treatment (NDT) or the Bobath approach) [21]. Therapeutic intervention of Rood’s approach for facilitation of tone involves 1) mobilizing touch and tactile stimulation, 2) brushing against the skin, 3) quick icing (if the patient tolerates it), 4) quick stretching, 5) tapping on the belly and tendon, 6) rubbing the skin to stimulate receptors below the skin, 7) heavy joint compression, 8) rolling, 9) and neutral warmth can help in decreasing the muscle tone that is abnormally high [22].

Neuromuscular electrical stimulation (NMES) is commonly used to treat pain, muscle strengthening, and sensorimotor rehabilitation. NMES can be used to induce muscle contractions and/or activate sensory pathways via "motor" and "sensory stimulation" that generate depolarization of the peripheral motor neuron, typically at the neuromuscular junction or motor end plate [23]. In acute and subacute critical illness neuropathy, as well as chronic diseases with concomitant muscle atrophy or weakness, NMES or direct muscle stimulation may be indicated. ES creates an electrical field between the electrodes, which causes depolarization of a neuron's cell membrane or, in the case of direct muscle stimulation, the elicitation of muscle fiber action potentials [24]. Even in chronic circumstances after GBS with maintained deep tendon reflexes, NMES can lead to long-term improvements in the motor tone of both extremities [23,24].

## Case presentation

Patient information

A 21-year-old female resident of Gadchiroli presented with a chief complaint of fever persisting for the past 10 days. She reported a history of vomiting a week ago, accompanied by an episode of dizziness and a subsequent fall eight days prior to the presentation. Additionally, the patient experienced a seizure episode eight days back following a traumatic event. Notably, there is a history of altered consciousness reported within the last three days. The patient denies any known comorbidities.

Clinical findings

The patient was observed in a supine position, and her level of consciousness was stupor. The Glasgow Coma Scale (GCS) score was 6/15. Mechanical ventilation was initiated in the continuous mandatory ventilation (CMV) mode, with positive end-expiratory pressure (PEEP) of 5 cm H_2_O and the fraction of inspired oxygen (FiO_2_) set at 30%. On auscultation, expiratory crackles were noted over the middle zone, with greater prominence on the left side. Motor examination revealed decreased tone, indicative of hypotonia, i.e., grade 1+ on the tone grading scale (TGS), in both the upper and lower limbs. Deep tendon reflexes are shown in Table 1. In Table 2, manual muscle testing grades according to the Medical Research Council (MRC) are given. The patient exhibited total dependence, highlighting the severity of the clinical presentation. On Brighton's diagnostic criteria for GBS, it was level 1. 

**Table 1:** Deep tendon reflexes +: Diminished reflex

Reflexes	Biceps jerk	Triceps jerk	Supinator jerk	Knee jerk	Ankle jerk	Plantar response
Right	Absent	+	Absent	Absent	Absent	Mute
Left	+	+	Absent	Absent	Absent	Mute

**Table 2:** Pre-intervention manual muscle testing Grade 0: No contraction

Muscle group	Right	Left
Shoulder flexors	0/5	0/5
Shoulder extensors	0/5	0/5
Elbow flexors	0/5	0/5
Wrist flexors	0/5	0/5
Wrist extensors	0/5	0/5
Hip flexors	0/5	0/5
Hip extensors	0/5	0/5
Knee flexors	0/5	0/5
Ankle plantar flexors	0/5	0/5
Ankle dorsiflexors	0/5	0/5

Clinical diagnosis

**Figure 1:**
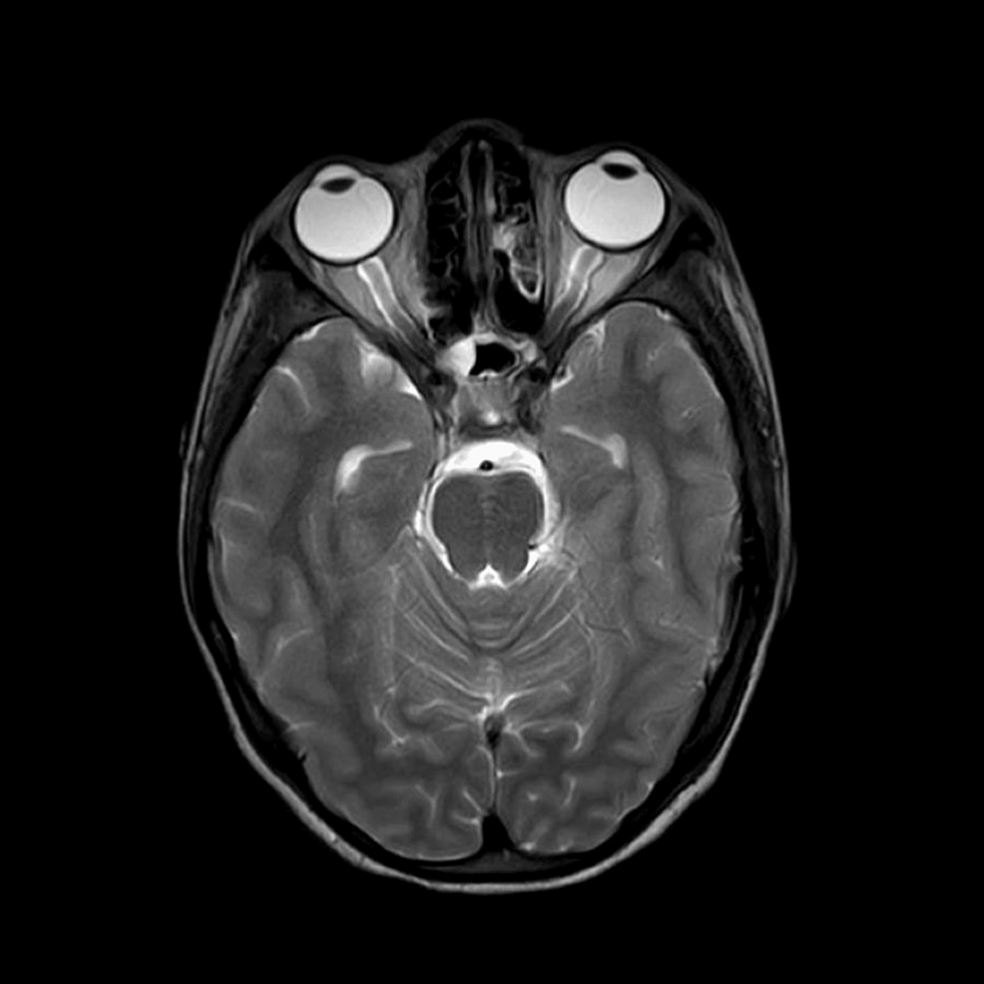
MRI The circle shows hyperintensity noted (right > left).

The patient underwent investigations like MRI, CBC, EEG, nerve conduction velocity (NCV), and CSF examination. The MRI study depicts, as shown in Figure 1, hyperintensity noted in bilateral mastoid air cells (right > left). EEG study showed rhythmic synchronous admixed alpha beta wave activity in the bilateral hemisphere in 7.5 microvolts. Intermittent sharp and high-amplitude waves and burst suppression were seen during the recording. NCV study revealed motor axonal polyneuropathy. CSF examination showed approximately 0.5 mL of whitish, translucent fluid in a clot activator bulb labeled CSF. After all the investigations and the clinical findings, the patient was diagnosed with autoimmune encephalopathy with GBS.

Physiotherapy intervention

A tailored physiotherapy protocol was implemented for two weeks, with the primary goal of improving patient consciousness and preventing secondary complications. Sensory stimulation techniques, such as light tapping and auditory stimulation, were used to enhance consciousness. Pressure sore prevention requires appropriate skin care through placement; caregivers were encouraged to provide proper positioning and to change positions every two hours. To improve vital capacity and prevent complications, respiratory challenges, such as decreased chest compliance and air entry, are addressed using chest physiotherapy techniques such as percussion, vibrations, and suctioning, as well as breathing exercises, coughing techniques, and incentive spirometry.

After that, physiotherapy rehabilitation, mainly focused on muscle weakness and hypotonia by using Rood's facilitatory approaches along with NMES, was planned for another one month. Physiotherapy care was provided once a day. Rood's facilitatory approaches, such as brushing against the skin, quick icing, tapping on the belly and tendon, and rubbing the skin to stimulate receptors below the skin on both upper and lower limbs, are performed for 10-20 seconds for four to five times per set. Heavy joint compression for shoulder, elbow, hip, knee, and ankle joints with 10-15 seconds hold for five times per set. Quick stretch to distal joints while performing passive range of motion (ROM) exercises for the upper and lower limbs (10 repetitions × 1 set). Administered NMES to flexor and extensor muscle groups of both upper and quadriceps, hamstrings, and calf muscles of both lower limbs, focusing on the progression from low-frequency stimulation for muscle contraction to higher frequencies for strength improvement. The frequency is set at 30 pulses per second. The pulse duration (measured in microseconds) for upper limb flexor and extensor muscles is generally 150-200, while for hamstrings, quadriceps, and calf muscles, it ranges from 200 to 300. Each muscle group received a 10-minute treatment. To address muscle weakness, the interventions start with active-assisted ROM exercise, progress to active ROM, and lastly, resisted ROM exercise. Proprioceptive neuromuscular facilitation (PNF) rhythmic initiation for upper and lower limb (D1 and D2 patterns) (10 reps × 1 set), strengthening exercises for upper and lower extremities (started with 1 kg of weight, progressing to 2 kg by the next 15 days with 10 repetition × 1 set). With NMES, higher frequencies are needed for strength improvement in the upper and lower limbs.

**Figure 2:**
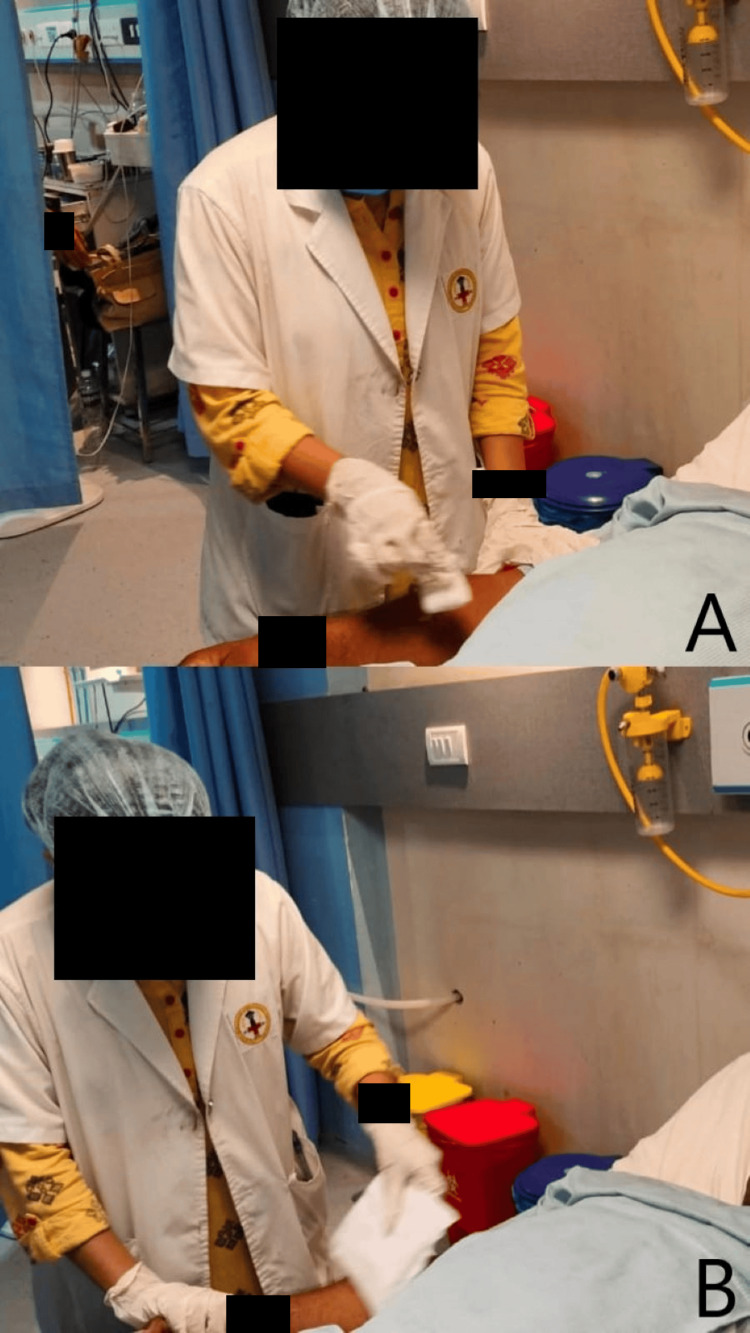
Therapist giving Rood's approach (A) Rubbing the skin. (B) Quick icing to the upper limb.

**Figure 3:**
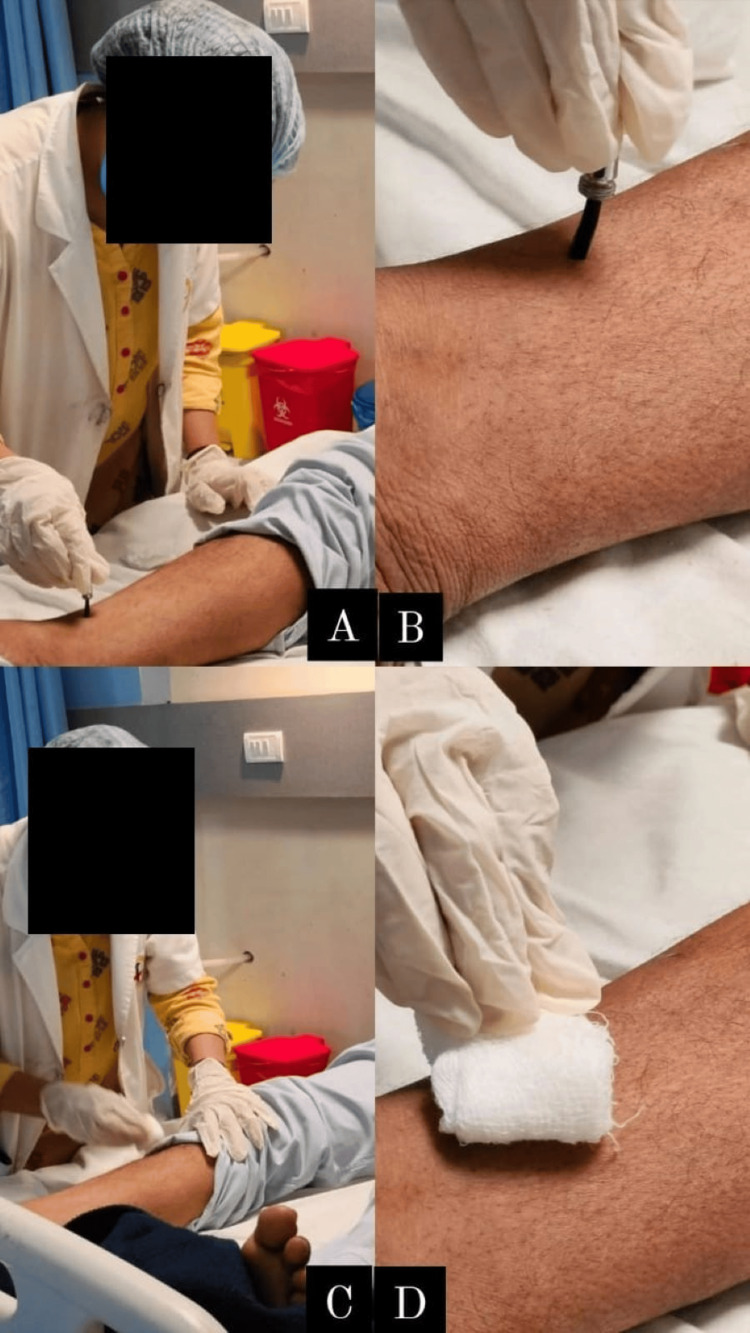
Therapist doing brushing and rubbing (Rood's approach) (A,B) Brushing against the skin. (C,D) Rubbing the skin to the lower limb.

The physiotherapy intervention received by the patient is summarized in Figure 2A, which shows the therapist using Rood's approach of rubbing the skin, and Figure 2B shows quick icing to the upper limb. Figures 3A-3B show brushing against the skin, and Figures 3C-3D show rubbing the skin to the lower limb.

Follow-up and outcome measures

A structured physiotherapy intervention regimen was initiated. Two weeks after the neuro-physiotherapy intervention, including multimodal stimulation, the patient gained consciousness. The chest was clear, and crackles were reduced. There was an increase in muscle tone, and the patient was able to stand. The prognostic plan for the patient is to restore all the problems mentioned above in one month. The muscle tone improvement after rehabilitation, according to TGS, is grade 2+, i.e., normal. Deep tendon reflexes post-rehabilitation are shown in Table 3, and in Table 4, manual muscle testing grades according to MRC post-rehabilitation are given. The patient exhibited minimal dependence. The outcome measures pre- and post-rehabilitation are shown in Table 5.

**Table 3:** Post-rehabilitation deep tendon reflexes ++: Normal reflex

Reflexes	Biceps jerk	Triceps jerk	Supinator jerk	Knee jerk	Ankle jerk	Plantar response
Right	++	++	++	++	++	Flexion
Left	++	++	++	++	++	Flexion

**Table 4:** Post-rehabilitation manual muscle testing Grade 4: Full range of motion against gravity, minimal resistance Grade 5: Full range of motion against gravity, maximal resistance

Muscle group	Right	Left
Shoulder flexors	5/5	5/5
Shoulder Extensors	5/5	5/5
Elbow flexors	5/5	5/5
Wrist flexors	5/5	5/5
Wrist extensors	5/5	5/5
Hip flexors	4/5	4/5
Hip extensors	4/5	4/5
Knee flexors	4/5	4/5
Ankle plantar flexors	4/5	4/5
Ankle dorsiflexors	4/5	4/5

**Table 5:** Outcome measures GCS score 6 (i.e., less than 8) indicates a comatose state. GCS score signifies being fully awake. ICU Mobility Scale Grade 0: Nothing (lying in bed) Grade 10: Walking independently without gait aid Hughes scale Grade 5: Requiring assisted ventilation Grade 1: Minor symptoms or signs of neuropathy but capable of manual work/capable of running Tone grading scale for both upper and lower limb Grade 0: No response flaccidity Grade 2+: Normal response Functional Independence Measure Level 2: Maximum assistance Level 4: Minimal assistance GCS, Glasgow Coma Scale

Sr. no.	Outcome measures	Pre-score	Post-score
1	GCS	6/15	15/15
2	ICU mobility scale	0/10	10/10
3	Hughes scale	5	1
4	Tone grading scale	0	2+
5	Functional Independence Measure	Level 2	Level 4

## Discussion

Encephalitis is a brain inflammatory illness characterized by a change in mental state, seizures, or focal neurologic impairments, as well as CSF fluid inflammation and MRI findings ranging from normal to significant anomalies. Most types of AE cause changes in mood, behavior, and memory, as well as decreased consciousness and epileptic attacks, the degree and majority of particular clinical manifestations over others, as well as the presence of extra characteristics (such as movement disorders, major psychiatric indicators, faciobrachial dystonic seizures, hyponatremia, and diarrhea), can vary [4]. GBS, on the other hand, is an acute polyneuropathy featuring flaccid paralysis and sensory/autonomic nerve damage. Characterized by symmetrical weakening of the limbs and hyporeflexia or areflexia, the severity of which peaks after four weeks [12]. Infection with *Mycoplasma* has been linked to neurological problems that include meningitis, encephalitis, and GBS. These problems are thought to be caused by invasions of pathogens or autoimmune reactions [15].

This case study emphasizes the critical significance of motor learning principles and neuroplasticity in physiotherapeutic care aimed at improving tone and muscular weakness in a patient having AE and GBS. The use of Rood's facilitatory techniques, which are based on motor learning, was critical in generating targeted muscle responses. These treatments, which included tapping, rubbing, brushing, heavy compression, and rapid icing, were used carefully to move the patient from a state of flaccidity to an enhanced muscular tone. Rood's facilitatory techniques were repeated and task-specific, which corresponded with motor learning principles, promoting adaptive alterations in brain pathways to improve neuromuscular coordination [18]. NMES, which used electrical impulses to activate muscles, provided a controlled environment for the nervous system to adapt and improve muscle strength. The combined use of Rood's facilitative approaches and NMES aims to harness the brain's plasticity, allowing not only tone restoration but also strength gains in the damaged muscle regions [20].

This case report's combination approach emphasizes the dynamic interplay between motor learning, neuroplasticity, and personalized treatment approaches. The rehabilitation strategy attempted not only to address the immediate barriers given by AE and GBS but also to encourage long-term gains in tone and muscle strength by incorporating these principles into the physiotherapeutic plan.

## Conclusions

In this case report, we present the successful application of Rood's facilitative techniques combined with NMES to enhance tone and strength in an individual suffering from AE with concurrent GBS. The patient experienced significant neurological impairments due to their dual diagnoses, which posed challenges for traditional rehabilitation methods. However, through targeted interventions based on Rood's principles, along with NMES therapy, the patient demonstrated notable improvements in muscle activation, motor function, and overall quality of life.

This innovative approach highlights the potential benefits of integrating novel therapeutic strategies into conventional care plans for patients experiencing complex neurological conditions such as those encountered by our subject. Further research is warranted to explore the efficacy and applicability of these techniques across various populations and settings, aiming to optimize treatment outcomes and improve functional recovery for individuals facing similar challenges.
